# A Study of Correlations within the Dimensions of Lower Limb Parts for Personal Identification in a Sudanese Population

**DOI:** 10.1155/2014/541408

**Published:** 2014-10-19

**Authors:** Altayeb Abdalla Ahmed

**Affiliations:** ^1^Department of Basic Medical Sciences, College of Medicine, King Saud bin Abdulaziz University for Health Sciences, Mail Code 3127, P.O. Box 3660, Riyadh 11481, Saudi Arabia; ^2^Anatomy Department, Faculty of Medicine, University of Khartoum, P.O. Box 102, Khartoum 11111, Sudan

## Abstract

The presence of an isolated limb or limb parts from different individuals presents a major challenge for medicolegal investigators in establishing identification in cases of wars, mass disasters, and criminal assaults because different populations have different sizes and proportions. The measurement of lower limb dimensions showed a high success rate in establishing individual identity in terms of sex and stature in various populations. However, there is a paucity of data concerning the correlation within the lower limb parts. This study aims to assess the existence of relationships within lower limb parts and to develop regression formulae to reconstruct limb parts from one another. The tibial length, bimalleolar breadth, foot length, and foot breadth of 376 right-handed Sudanese adults were measured. The results showed that all variables were significantly larger in males than in females. A significant positive correlation (*P* < 0.001) was found within the lower limb parts. Sex-specific linear equations and multiple regression equations were developed to reconstruct the lower limb parts in the presence of single dimension or multiple dimensions from the same limb. The use of multiple regression equations provided a better reconstruction than simple regression equations. These results are significant in forensics and orthopedic reconstructive surgery.

## 1. Introduction

The human lower limb is structurally and functionally adapted for bipedal locomotion and weight bearing. This adaptation affects bone length and strength, joint complexes, muscle mass, and muscle origin and insertion in relation to lines of joints and foot development (e.g., arches) [1]. Moreover, humans have very long and strong lower limb bones relative to upper limb bones [1]. The human lower leg and foot have been the subjects of research in various nonforensic fields, such as anatomy, anthropology, evolution, ergonomics, and orthopedics. The study of the human lower leg and foot in forensics identification has been emphasized because of the increased likelihood of retaining the tibia (the second largest bone in the body) and the foot (often protected by the shoes) in the case of mass disasters, terrorists' attacks, wars, explosions, high-impact transportation accidents, and mass murders [2, 3].

The identification of a deceased person is the mainstay of forensic analysis. Therefore, the primary aim for any forensic anthropologist is to reconstruct an osteobiography consisting of a set of biological attributes, for example, sex, stature, and age [4]. However, reconstruction presents a major challenge in cases where there is presence of multiple isolated and commingled fleshed peripheral body parts, as in mass fatalities, or when the integrity of multiple bodies is compromised intentionally to conceal the identity of the victim. In these situations, the priority for medicolegal investigators is to sort and match these commingled parts to one another to determine the minimum number of subjects involved before making any attempt to establish meaningful identification [5].

Biological attributes have been determined from lower limb bones using direct/radiological measurements or morphological traits evaluation [2, 6, 7]. Anthropometry has been proven to be a reliable technique to measure body parts [8]; various body parts have been measured to develop population-specific standards for both living and unidentified deceased subjects, with high success rates [9, 10]. The foot and the lower leg have been shown to be a relatively accurate biological characteristic from which identification can be made in terms of estimating sex and stature [11, 12].

The final shape of one's skeleton is governed by balanced interaction between genetics and environment throughout life [13]. Body proportions are variable between populations [14]; intralimb indices have been studied to assess the effect of environmental factors on skeletal growth. However, an extensive literature review reveals a scarcity in assessment of intralimb parts correlation in forensics. To the best of my knowledge, the only reported data studied the correlation within and between foot and hand dimensions for personal identification in endogamous Indian castes (i.e., Rajputs) [15]. The paucity of published literature and the findings of significant correlation between hand and foot length led to the recommendation that similar studies be conducted using different parts among different populations [15].

The reconstruction of lower intralimb parts has many advantages. It can be used in the process of matching multiple mutilated or dismembered fleshed lower limb parts, which is the initial step for any identification conducted by forensic investigators. Estimation of part lengths from identical or different breadths can provide a tool for stature estimation. Reconstruction can also be used in post-traumatic reconstructive surgeries to estimate the part's length or breadth. Hence, this study aims to explore the correlations within lower limbs and to develop population-specific standards for the reconstruction of different parts. This research can provide valuable data to future comparative studies within other populations, especially where mixed Arabic and African genes predominate.

## 2. Materials and Methods

### 2.1. Sample

Measurements for the present study were taken from a sample consisting of 376 adult subjects (187 male and 189 female) recruited from Khartoum. The subjects were between 25 and 30 years of age: the mean age was 29.45 ± 1.52 years for males and 29.48 ± 1.54 years for females. The participants were required to sign a consent form and to complete a questionnaire that contained basic demographical data and general questions (e.g., handedness).

The participants were normal, right-handed, and healthy Sudanese Arab volunteers who comprise the majority of the demographics of contemporary Sudan. All persons with chronic illnesses, skeletal deformities, pathologies, surgeries, or fractures that could impede accurate lower limb measurements were excluded from this study. Ethical research approval was granted by the Ethical Committee of the Faculty of Medicine, University of Khartoum.

### 2.2. Methodology

Four lower limb measurements were taken from each subject using standard anthropometric instruments in units of centimeters and rounded to the nearest millimeter. These measurements were obtained from the subject's left side according to the procedure described by the International Biological Program [16, 17] in a well-lit room and repeated two times to record the means. These measurements included the parameters detailed below.

#### 2.2.1. Tibia Length

Tibia length was measured with a Harpenden anthropometer (Holtain Ltd., Crosswell, UK) on the subjects as they were seated with their knees in a semiflexed position. The measurements were taken as the distance from the most prominently palpable portion of the medial condyle of the tibia to the tip of medial malleolus [18].

#### 2.2.2. Bimalleolar Breadth

Bimalleolar breadth was measured using a digital sliding caliper (Mitutoyo, Aurora, IL, USA) as the distance between the most medial projection of the medial malleolus and the most lateral projection of the lateral malleolus [16].

#### 2.2.3. Foot Length

Foot length was measured with a Harpenden anthropometer as the straight distance between the most posterior and prominent part of the heel and the most distal part of the longest toe of the foot, as the subject stood upright with equal pressure on both feet [19].

#### 2.2.4. Foot Breadth

Foot breadth was measured using a Harpenden anthropometer as the straight distance between the metatarsal fibulare and the metatarsal tibiale, with the foot in a fully “loaded” position [20].

### 2.3. Technical Error of Measurement

Technical errors can significantly affect accuracy and reliability; therefore, the instruments were checked regularly for accurate readings. Additionally, before beginning the primary data collection, a pilot study was conducted to assess the precision and reliability of acquiring the lower limb measurements. The lower limb dimensions of the same 15 subjects were measured on two different evaluation days, with two days between the remeasurements. The relative technical error of measurement (rTEM) and the coefficient of reliability (*R*) were calculated from the two sets of measurements [21, 22]. The intraobserver measurement error and reliability were calculated to be within the acceptable standards for all measurements (*R* > 0.9; rTEM < 5%) [21].

### 2.4. Statistical Analysis

The data were analyzed using the Statistical Package for the Social Sciences (SPSS), version 14 (SPSS, Inc., Chicago, IL). Means, standard deviations, and ranges were used to summarize the anthropometric measurements. An independent *t*-test was used to test the differences between males and females mean measurements. Pearson's correlation coefficients were calculated to find the correlation between various measurements of the lower limb. Regression is the determination of the statistical relationship between two or more variables [23]. Sex-specific simple and multiple regression equations for the estimation of different parts were developed using the lower limb measurements. A *P* value of less than 0.05 was considered significant. The accuracy of these equations was validated by the obtained standard error of estimate (SEE). A low SEE indicated a higher accuracy.

## 3. Results

The descriptive statistics for the lower limb dimensions in males and females are presented in Table 1. The four dimensions were higher for males than females. Statistically significant sex differences were observed for all measurements (*P* < 0.001). These results indicated the presence of sexual dimorphism in the study sample; hence, sex-specific correlation coefficients were calculated, and regression equations were developed.

Table 2 shows that the four lower limb dimensions were significantly correlated with one another in both sexes (*P* < 0.001). The highest correlation coefficient in both sexes was observed between the tibial and foot length (*r* = 0.649 for males, *r* = 0.611 for females). Moreover, a high correlation coefficient was observed between foot length and breadth (*r* = 0.570 for males, *r* = 0.547 for females).

Several possible sex-specific simple linear regression equations and SEE values were derived for the reconstruction of each lower limb measurement from any possible single measurements (Tables 3 and 4). The regression coefficients were observed to be statistically significant in all derived equations. The minimum SEE value for the construction of tibial lengths for both sexes was obtained using foot lengths (±1.722 cm in males and ±1.532 cm in females). The bimalleolar breadths were reconstructed with minimum SEE value using foot lengths for both sexes (SEE ± 0.354 cm in males and ±0.255 cm in females). The minimum SEE value for the equations reconstructing foot lengths was derived using tibial lengths for both sexes (SEE ± 0.967 cm in males and ±0.826 cm in females). By contrast, foot breadths were reconstructed with the minimum SEE value using bimalleolar breadths among males (SEE ± 0.566 cm) and foot lengths among females (±0.440 cm).

Multiple regression equations were formulated to evaluate whether the accuracy of the reconstruction of the lower limb dimension can be improved using multiple variables, as shown in Tables 5 and 6. When stepwise analysis was used, the tibial length was best predicted using the foot length only in both sexes. Additionally, the equations reconstructing bimalleolar breadths, foot lengths, and foot breadths utilized the same variables in both sexes. The coefficient of determination increased in multiple stepwise regressions equations when compared to the single regression equations. The multiple direct regression equations revealed lower SEE for both sexes compared with single direct or stepwise regression, except for the tibial length (Table 6).

## 4. Discussion

In this study, lower limb dimensions are found to be sexually dimorphic. This result is in accordance with earlier observations among different populations in which males have consistently larger dimensions when compared to females [24–26]. Earlier studies among Sudanese showed that among lower limb dimensions, breadth dimensions are more sexually dimorphic than length dimensions [11]. The means of the four lower limb dimensions used in this study were found to be different from those reported in earlier studies among different populations. This finding agrees with previous reports among Sudanese Arabs and other populations, where these differences have been attributed to factors such as genetic background, nutrition, climate, and levels of physical activity [27–29]. Moreover, different races and geographical areas show different proportions between different bones [30]. Hence, the correlations between intralimb parts may vary accordingly, which requires a specific standard for each population [31].

The correlation coefficients within lower limb parts were highly significant (*P* < 0.001) and positively correlated. This finding indicates the potential for reconstructing the measurements of one part from another dimension. The highest correlation was observed to be between tibial and foot lengths. There is a paucity of studies regarding the correlation between and within limbs. Existing studies document the existence of significant correlation between the hand and foot length in Indians and Nigerians [32, 33]. The only study reporting a correlation within lower limb parts utilized the foot length and breadth in Indians (Rajputs) [15]; hence, the correlations degrees in this study could not be compared for many of the limb dimensions. The findings of this study confirm the previous assumption that different intralimb parts are well correlated to one another [15]. The highest correlation between tibial length and breadth dimensions was among females, whereas males showed higher correlation between tibial and foot length. Correlation between bimalleolar breadth and foot measurements, however, was the highest among males. Additionally, the correlation between foot length and breadth was higher among males. This result concurs with the previous finding in North Indians (Rajputs), in whom the males showed higher correlation between foot length and foot breadth [15]. These findings can be attributed to the existence of sexual differences in body proportions and that different parts within the same limb do not exhibit perfectly parallel variations. Moreover, the development of intralimb proportions is affected by one's population affinity and ecogeographic differences (e.g., climate and nutrition) [34].

Linear regression models were derived to estimate the intralimb parts from one another. The best predictor of tibial length was foot length in both sexes (SEE ± 1.722 for males and 1.532 for females). Foot length is best predicted by utilizing tibial length in both sexes (0.967 for males and 0.826 for females). Bone lengths are found to be attributed primarily to genetics rather than to health and nutrition [27]. However, the bimalleolar breadth was predicted more accurately when utilizing the foot length in both sexes. The breadth of epiphysis is affected by both genetics and environmental factors [35]. In females, the best predictor for foot breadth was foot length with a SEE of ±0.440, compared with males, in which the best predictor was the bimalleolar breadth with a SEE of ±0.566. This variability can be attributed to environmental and lifestyle factors affecting the foot, such as working patterns or types of shoes being worn. The findings of this study predicting foot length from foot breadth (±0.873–1.044) were comparable to the results obtained by Kanchan et al. [15] in endogamous cast (Rajputs) (SEE ± 0.904–1.042), indicating genetic similarity between Sudanese Arabs.

Multiple regression equations resulted in a better estimation of the body parts when compared with simple regression equations; thus, it is better to use multiple regression equations when possible. The stepwise analysis demonstrated an accuracy rate close to the direct approach. Females were found to have a lower SEE in all equations compared with males. This difference can be attributed to a wider variability in male size compared with similarity in female size.

The findings of this study demonstrate the possibility of reconstruction of lower limb parts from one another. This study has its limitations: the measurements used are from the left side only, although other studies on the right- and left-sided dominance in tibia and foot dimensions are inconclusive. It would be optimal to investigate both sides. Additionally, these models are applicable only to Sudanese Arabs.

## 5. Conclusion

Limb proportions can vary among populations, depending upon various factors such as biological affinity, growth, life conditions, and lifestyle. This study is the first to evaluate reconstruction from intralower limb parts from a mixed ethnic adult population (i.e., Arab individuals with mixed African genes). The significance of this study lies principally in its applicability and accuracy in the reconstruction of body parts from one another, which can be useful in the presence of multiple commingled parts during forensic and medicolegal examination and orthopedics, as well as in comparative studies. This study also shows a positive and statistically significant correlation between lower limb parts in both sexes. The predictive accuracy of multiple regression models is better than that of simple linear regression models.

## Figures and Tables

**Table 1:** Descriptive statistics for lower limb dimensions (in cm) in both sexes.

Parameter	Males				Females				Independent *t*-test	
	Mean	SD	Min	Max	Mean	SD	Min	Max	*t*- value	*P* (2-tailed)
Tibial length	41.66	2.26	36.20	47.60	38.21	1.93	33.10	42.40	15.925	∗∗∗
Bimalleolar breadth	6.83	0.46	5.80	7.80	5.75	0.32	5.10	6.50	26.285	∗∗∗
Foot length	26.53	1.27	23.70	30.00	24.11	1.04	21.30	26.4	20.192	∗∗∗
Foot breadth	9.70	0.73	7.10	11.30	8.40	0.52	7.10	9.70	19.819	∗∗∗

SD: standard deviation.

∗∗∗The *t*-test was significant at the 0.001 level (2-tailed).

**Table 2:** Pearson correlation within the lower limb dimensions (in cm).

Parameter	Males				Females			
	TL	BMB	FL	FB	TL	BMB	FL	FB
TL	—	0.301∗∗∗	0.649∗∗∗	0.341∗∗∗	—	0.388∗∗∗	0.611∗∗∗	0.362∗∗∗
BMB	0.301∗∗∗	—	0.643∗∗∗	0.635∗∗∗	0.388∗∗∗	—	0.595∗∗∗	0.542∗∗∗
FL	0.649∗∗∗	0.643∗∗∗	—	0.570∗∗∗	0.611∗∗∗	0.595∗∗∗	—	0.547∗∗∗
FB	0.341∗∗∗	0.635∗∗∗	0.570∗∗∗	—	0.362∗∗∗	0.542∗∗∗	0.547∗∗∗	—

TL: tibial length, BMB: bimalleolar breadth, FL: foot length, and FB: foot breadth.

∗∗∗The *P* value was significant at the 0.001 level (two-tailed).

**Table 3:** Linear regression equations for reconstruction of lower limb dimensions (in cm) in males.

Regression equation	*R*	*R* ^2^	±SEE	*P* value
TL = 31.578 + 1.477 × BMB	0.301	0.091	±2.159	∗∗∗
TL = 11.000 + 1.156 × FL	0.649	0.421	±1.722	∗∗∗
TL = 31.434 + 1.054 × FB	0.341	0.116	±2.128	∗∗∗
BMB = 4.263 + 0.062 × TL	0.301	0.091	±0.441	∗∗∗
BMB = 0.631 + 0.234 × FL	0.643	0.413	±0.354	∗∗∗
BMB = 2.945 + 0.400 × FB	0.635	0.403	±0.357	∗∗∗
FL = 11.341 + 0.364 × TL	0.649	0.421	±0.967	∗∗∗
FL = 14.456 + 1.768 × BMB	0.643	0.413	±0.974	∗∗∗
FL = 16.927 + 0.990 × FB	0.570	0.325	±1.044	∗∗∗
FB = 5.098 + 0.110 × TL	0.341	0.116	±0.689	∗∗∗
FB = 2.828 + 1.006 × BMB	0.635	0.403	±0.566	∗∗∗
FB = 0.978 + 0.329 × FL	0.570	0.325	±0.602	∗∗∗

TL: tibial length, BMB: bimalleolar breadth, FL: foot length, FB: foot breadth, *R*: correlation coefficient, *R* ^2^: coefficient of determination, and SEE: standard error of estimate.

∗∗∗The *P* value was significant at the 0.001 level (two-tailed).

**Table 4:** Linear regression equations for reconstruction of lower limb dimensions (in cm) in females.

Regression equation	*R*	*R* ^2^	±SEE	*P* value
TL = 24.588 + 2.368 × BMB	0.388	0.150	±1.784	∗∗∗
TL = 10.882 + 1.134 × FL	0.611	0.373	±1.532	∗∗∗
TL = 27.021 + 1.333 × FB	0.362	0.131	±1.804	∗∗∗
BMB = 3.330 + 0.063 × TL	0.388	0.150	±0.292	∗∗∗
BMB = 1.393 + 0.181 × FL	0.595	0.354	±0.255	∗∗∗
BMB = 3.009 + 0.327 × FB	0.542	0.249	±0.266	∗∗∗
FL = 11.542 + 0.329 × TL	0.611	0.373	±0.826	∗∗∗
FL = 12.837 + 1.959 × BMB	0.595	0.354	±0.838	∗∗∗
FL = 15.003 + 1.084 × FB	0.547	0.299	±0.873	∗∗∗
FB = 4.645 + 0.098 × TL	0.362	0.131	±0.490	∗∗∗
FB = 3.224 + 0.899 × BMB	0.542	0.294	±0.442	∗∗∗
FB = 1.757 + 0.275 × FL	0.547	0.299	±0.440	∗∗∗

TL: tibial length, BMB: bimalleolar breadth, FL: foot length, FB: foot breadth, *R*: correlation coefficient, *R* ^2^: coefficient of determination, and SEE: standard error of estimate.

∗∗∗The *P* value was significant at the 0.001 level (two-tailed).

**Table 5:** Stepwise regression equations for reconstruction of lower limb dimensions (in cm).

Sex	Regression equation	*R*	*R* ^2^	±SEE	*P* value
Male	TL = 11.000 + 1.156 × FL	0.649	0.421	±1.722	<0.001
	BMB = 0.386 + 0.151 × FL + 0.251 × FB	0.721	0.520	±0.321	<0.001
	FL = 5.574 + 0.281 × TL + 1.353 × BMB	0.801	0.641	±0.764	<0.001
	FB = 1.143 + 0.928 × BMB + 0.053 × TL	0.654	0.428	±0.556	<0.001

Female	TL = 10.882 + 1.134 × FL	0.611	0.373	±1.532	<0.001
	BMB = 1.065 + 0.130 × FL + 0.186 × FB	0.649	0.421	±0.242	<0.001
	FL = 6.917 + 0.241 × TL + 1.389 × BMB	0.724	0.524	±0.721	<0.001
	FB = 2.033 + 0.784 × BMB + 0.048 × TL	0.567	0.321	±0.434	<0.001

TL: tibial length, BMB: bimalleolar breadth, FL: foot length, FB: foot breadth, *R*: correlation coefficient, *R* ^2^: coefficient of determination, and SEE: standard error of estimate.

**Table 6:** Multiple direct regression equations for reconstruction of lower limb dimensions (in cm).

Sex	Regression equation	*R *	*R* ^2^	±SEE	*P* value
Male	TL = 11.130 + 1.199 x FL − 0.133 × FB	0.650	0.423	±1.725	<0.001
	FL = 5.271 + 0.267 × TL + 1.107 × BMB + 0.265 × FB	0.809	0.654	±0.752	<0.001
	FB = 0.360 + 0.014 × TL + 0.738 × BMB + 0.140 × FL	0.670	0.449	±0.547	<0.001

Female	TL = 10.623 + 1.093 × FL + 0.148 × FB	0.612	0.374	±1.535	<0.001
	FL = 5.991 + 0.219 × TL + 1.031 × BMB + 0.456 × FB	0.748	0.560	±0.695	<0.001
	FB = 0.890 + 0.009 × TL + 0.555 × BMB + 0.165 × FL	0.610	0.372	±0.419	<0.001

TL: tibial length, BMB: bimalleolar breadth, FL: foot length, FB: foot breadth, *R*: correlation coefficient, *R* ^2^: coefficient of determination, and SEE: standard error of estimate.
